# A mixed-method evaluation of the relationship between Oxford classification scores and longitudinal changes in proteinuria in patients with immunoglobulin A nephropathy

**DOI:** 10.3389/fendo.2022.890900

**Published:** 2023-01-10

**Authors:** Ri-Cong Xu, Jian-Ying Guo, Tao Cao, Yi Xu, Ying Liao, Yu-Na Chen, Hai-Ying Song, Xiao-Jie Chen, Mi-Jie Guan, Fei Tang, Qiong Xiang, Xing-Lin Chen, Qi-Jun Wan

**Affiliations:** ^1^ Department of Nephrology, The First Affiliated Hospital of Shenzhen University, Shenzhen, China; ^2^ Department of Nephrology, The Second People's Hospital of Shenzhen, Shenzhen, China; ^3^ Department of Epidemiology and Biostatistics, Empower U, X&Y solutions Inc., Boston, MA, United States

**Keywords:** IgA nephropathy, Oxford classification, proteinuria/creatinine ratio, mixed methods, renal function

## Abstract

**Introduction:**

This study aimed to investigate the relationship between Oxford Classification scores and longitudinal changes in proteinuria in patients with immunoglobulin A nephropathy (IgAN).

**Methods:**

The study was a single-center retrospective cohort study involving 358 patients with primary IgAN who were treated at the Shenzhen Second People’s Hospital, China, between January 2011 and May 2021. Multivariate linear regression and generalized additive mixed models (GAMMs), adjusted for traditional risk confounders, were used to evaluate the correlation between scores for mesangial hypercellularity (M), endocapillary hypercellularity (E), segmental glomerulosclerosis (S), tubular atrophy/interstitial fibrosis (T), and crescents (C) (known as the Oxford Classification MEST-C score system), with proteinuria/creatinine ratio (PCR) at the time of renal biopsy and longitudinal changes in PCR, respectively.

**Results:**

The median PCR was 1061 mg/g, and it increased on average by 68.82 mg/g per year in these patients. Among patients with renal insufficiency, compared with patients without relative lesions, those with E present (E1) (1153.44; 95% confidence interval [CI], 188.99–2117.89 mg/g) and C > 0 (C1/2) (1063.58; 95% CI, 185.25–1941.90 mg/g) were associated with increased PCR levels at the time of renal biopsy. What’s more, S present (S1) (194.96; 95% CI, 54.50–335.43 mg/g per year) was associated with the fastest PCR increase; C > 0 (C1/2) (147.59; 95% CI, 8.32–286.86 mg/g per year) and T >25% (T1/2) (77.04; 95% CI, 7.18–146.89 mg/g per year), were also correlated with a faster PCR increase. In patients with normal kidney function, associations between S1 (55.46; 95% CI, 8.93–101.99 mg/g per year) and E1 (94.02; 95% CI, 21.47–166.58 mg/g per year) and PCR change could be observed. Additionally, in patients with overweight/obesity, S1 (156.09; 95% CI, 52.41–259.77 mg/g per year), E1 (143.34; 95% CI, 35.30–251.38 mg/g per year), T1/2 (116.04; 95% CI, 22.58–209.51 mg/g per year), as well as C1/2 (134.03; 95% CI, 41.73–226.32 mg/g per year) were associated with noticeably quicker PCR increase.

**Conclusions:**

Overall, E1 and C1/2 were independently associated with raised proteinuria levels at the time of renal biopsy, and S1, E1, T1/2, C1/2 were independently associated with a longitudinal increase in proteinuria in the patients with IgAN, especially in those with renal insufficiency or overweight/obesity, suggesting that currently available treatments might not be satisfactory, and weight control might be beneficial. Individual therapy development might benefit from the use of the Oxford Classification system.

## Introduction

Immunoglobulin A nephropathy (IgAN) is the most common form of primary glomerulonephritis and the leading cause of end-stage renal disease in China (1). Variable clinical manifestations, from isolated microscopic hematuria to severe renal function decline, can be seen in patients with IgAN, and there is worldwide consensus regarding the use of the Oxford Classification system to evaluate the severity of renal damage. This system uses the following markers: mesangial hypercellularity (M), endocapillary hypercellularity (E), segmental glomerulosclerosis (S), and tubular atrophy and interstitial fibrosis (T), and the revised Oxford Classification, called the MEST-C score system, also includes the presence of crescents (C) (2). Of these five markers, T lesions have been consistently confirmed to predict renal disease progression, with more variable results for M, E, S, and C lesions (3–18). Repeated measurements of the estimated glomerular filtration rate (eGFR) have shown that after renal biopsy, patients with S present (S1), T >50% (T2), and C >25% glomeruli (C2) lesions have notably faster renal function decline (19).

Factors such as renal injury, massive proteinuria, hypertension, and a low GFR, which could increase the risk of entering end-stage renal disease (ESRD) by 10–15 times, have been found to be present in more than half of patients with IgAN (20–22). However, the only risk factor currently considered in the kidney disease: Improving Global Outcomes guidelines, with respect to targeting corticosteroid and/or immunosuppressive treatment in IgAN, is proteinuria at a persistent level of >0.75–1 g/day, with three months of optimized supportive care (23). At present, there is insufficient evidence to support the use of Oxford Pathological Classification MEST-C scores to determine whether immunosuppressive therapy would benefit patients with IgAN, as most published randomized controlled trials to date (24–27) have not considered renal pathology at enrollment.

Thus, in this study, linear regression and mixed methods are used to evaluate the association of each Oxford Classification score (MEST-C) with baseline and longitudinal changes in proteinuria respectively in patients with IgAN.

## Results

**Figure 1:**
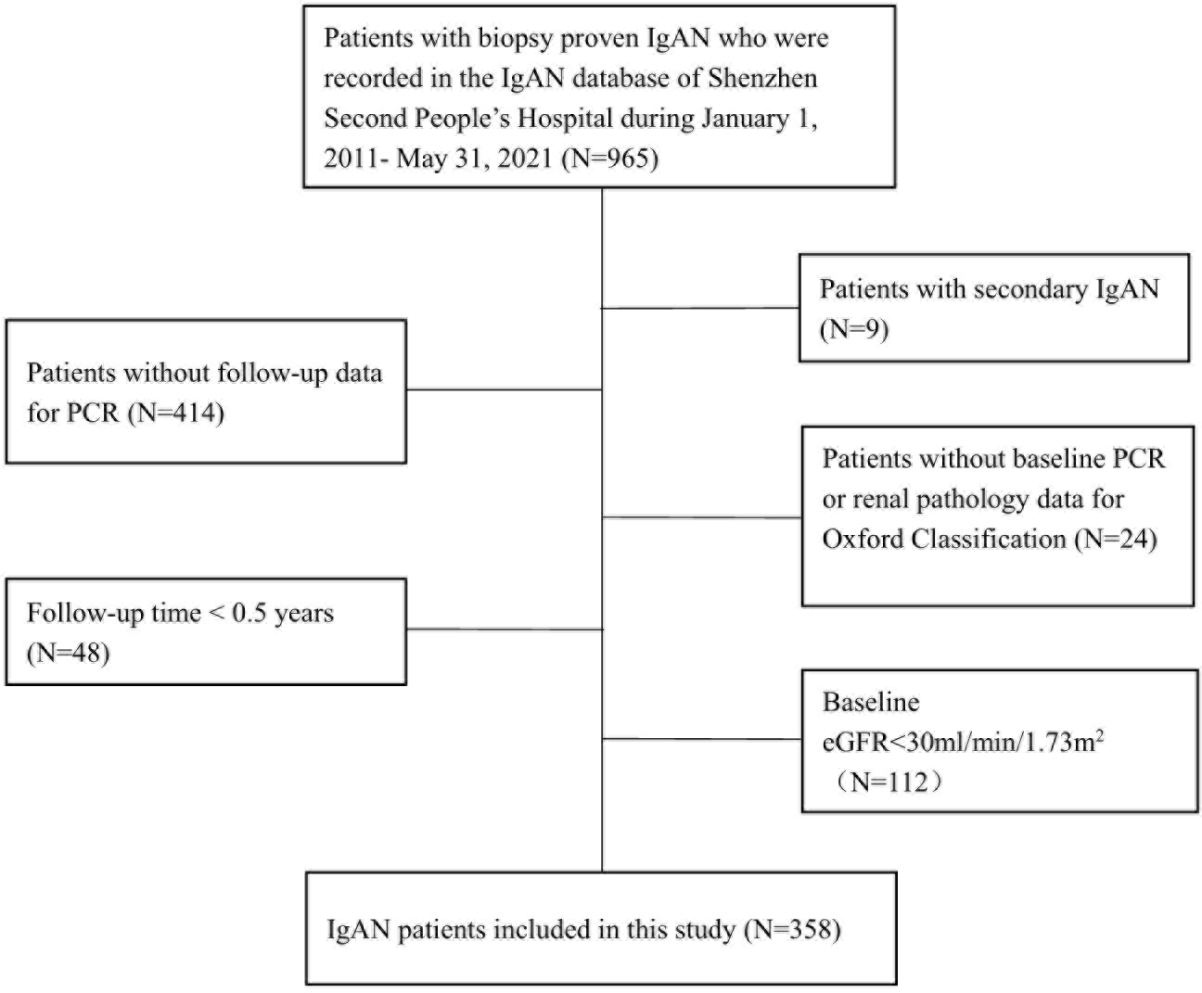
Flowchart of patients with immunoglobulin A nephropathy included in the study.

A total of 358 patients were included in the study (**Figure 1**). The mean age was 34.94 ± 9.43 years, 49.16% of the patients were male, the mean BMI was 22.73 ± 3.40 kg/m^2^, 43.12% of them were overweight/obesity, and 71.23% of them with hematuria at the time of renal biopsy. With regard to the Oxford Classification system, in each score group, M >0.5 (M1), E present (E1), and S1 lesions accounted for 76.84%, 22.60%, and 33.15% of all lesions, respectively, and T >25% (T1/2) and C > 0 (C1/2) lesions accounted for 23.66% and 54.86% of all lesions, respectively. (**Table 1**) The characteristics of patients with different Oxford Classification scores were also shown (**Supplementary Tables 1–5**).

**Table 1:** Baseline characteristics and pathological features of IgAN patients.

Variables	Total
Age, yr	34.94 ± 9.43
Male, n (%)	176 (49.16%)
BMI, kg/m^2^	22.73 ± 3.40
BMI group, n (%)	
Underweight/normal weight	186 (56.88%)
Overweight/obesity	141 (43.12%)
SBP, mmHg	127.51 ± 18.82
DBP, mmHg	82.34 ± 12.67
MAP, mmHg	96.42 ± 13.71
Hemoglobin, g/L	128.82 ± 19.73
Albumin, g/L	39.24 ± 5.35
Total cholesterol, mmol/L	4.81 ± 1.31
Triglyceride, mmol/L	1.59 ± 1.26
Uric acid, umol/L	403.61 ± 110.75
eGFR, ml/min/1.73m^2^	81.48 (58.73, 101.78)
Proteinuria/creatinine ratio, mg/g	1061.00 (603.00, 1778.00)
Hematuria, n (%)	255(71.23%)
Follow up time, yr	2.60 (1.30, 5.05)
Treatments, n (%)	
RASi alone, n (%)	146 (43.07%)
RASi + CSs/ISs, n (%)	112 (33.04%)
CSs/ISs alone, n (%)	47 (13.86%)
No RASi and CSs/ISs, n (%)	34 (10.03%)
Oxford Classification, n (%)	
Mesangial hypercellularity (M1)	272 (76.84%)
Endocapillary hypercellularity (E1)	80 (22.60%)
Segmental glomerulosclerosis (S1)	118 (33.15%)
Tubular atrophy/interstitial fibrosis (T1/2)	84 (23.66%)
Cresent (C1/2)	192 (54.86%)

Data presented as mean ± SD, median (25th, 75th) or number (percent).

SBP, systolic blood pressure; DBP, diastolic blood pressure; MAP, mean artery pressure; eGFR, estimated glomerular filtration rate; BMI, body mass index; RASi, renin-angiotensin system inhibitors; CSs/ISs, corticosteroids and (or) immunosuppressants.

The median eGFR was 81.48 ml/min/1.73 m^2^ (interquartile range [IQR], 58.73–101.78 ml/min/1.73 m^2^), the median proteinuria/creatinine ratio (PCR) was 1061 mg/g (IQR, 603–1778 mg/g). (**Table 1**) Using a two-piecewise linear regression model, after adjusting for age, gender, mean arterial pressure (MAP), body mass index (BMI) and the Oxford Classification MEST-C markers, we discovered that the relationship between eGFR and proteinuria was non-linear (**Supplementary Figure 1**), and an inflection point of 94.3ml/min/1.73m^2^ was found (**Supplementary Table 6**). On the left of the inflection point, eGFR was negatively associated with proteinuria [β -16.18, 95% CI (-32.22)-(-0.14), p=0.048]. On the right of the inflection point, the correlation between eGFR and proteinuria was not significant (p > 0.05).

**Figure 2:**
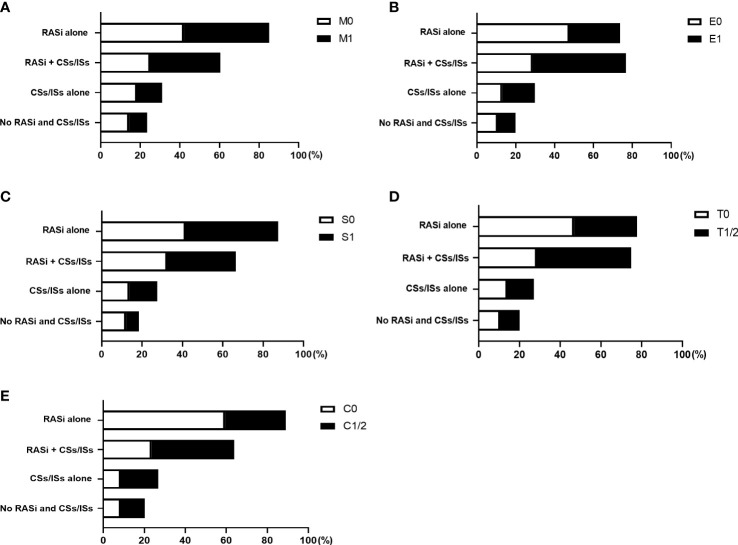
Treatments of patients with different Oxford Classification MEST-C scores. The proportion of patients took renin-angiotensin system inhibitors (RASi) alone, RASi in combination with corticosteroids and (or) immunosuppressants (CSs/ISs), CSs/ISs alone, as well as no RASi and CSs/ISs, in patients with and without M **(A)**, E **(B)**, S **(C)**, T **(D)**, C **(E)** lesions were shown.

After renal biopsy, 43.07% of the patients took renin-angiotensin system inhibitors (RASi) alone, 33.04% of them took RASi in combination with corticosteroids and (or) immunosuppressants (CSs/ISs), 13.86% of them took CSs/ISs alone, and 10.03% of patients took neither RASi or CSs/ISs. (**Table 1**) Treatments divided by different Oxford Classification scores were shown in **Supplemental Tables 1–5** and **Figure 2**. Overall, patients with active lesions including E1 (E1, 64.93%; E0, 41.61%) and C1/2 (C1/2: 58.6%; C0, 32.03%) were more likely to be treated with CSs/ISs.

At the time of renal biopsy, patients with E1, S1, T1/2, C1/2 lesions exhibited higher levels of proteinuria than patients without relative lesions (p<0.05, **Supplementary Tables 1–5**). In order to investigate the effect of different Oxford Classification scores on baseline proteinuria, multivariate linear regression was used. In patients with renal insufficiency (30≤eGFR<60 ml/min/1.73m^2^), after adjusting for covariates including age, gender, MAP, eGFR, BMI and MEST-C score, we found E1 (1153.44; 95% CI, 188.99–2117.89 mg/g; p = 0.022) and C1/2 (1063.58; 95% CI, 185.25–1941.90 mg/g; p = 0.020) lesions were independently associated with a rise of PCR at the time of renal biopsy. However, no associations were found between M1, S1, T1/2 and PCR in the adjusted models (p > 0.05). To clarify whether these relationships existed in patients with normal kidney function (eGFR ≥60 ml/min/1.73 m^2^), subgroup analysis was performed, but the associations were not significant (p > 0.05) (**Table 2**).

**Table 2:** Association of the Oxford Classification score MEST-C with baseline PCR at the time of renal biopsy according to 30≤eGFR<60 and eGFR ≥60 ml/min/1.73m^2^.

Oxford Classification	30≤eGFR<60 ml/min/1.73m^2^		eGFR≥60ml/min/1.73m^2^	
	Beta (95% CI)	*P* value	Beta (95% CI)	*P* value
M1	6.16 (-1331.54, 1343.86)	0.993	-57.37 (-666.93, 552.19)	0.854
E1	1153.44 (188.99, 2117.89)	0.022	207.68 (-490.80, 906.17)	0.561
S1	-400.73 (-1294.84, 493.38)	0.382	433.75 (-176.75, 1044.24)	0.165
T1/2	296.30 (-677.91, 1270.51)	0.553	369.53 (-577.85, 1316.90)	0.445
C1/2	1063.58(185.25, 1941.90)	0.020	14.85 (-502.59, 532.28)	0.955

Linear regression models were used to investigate the associations of M, E, S, T, and C with the baseline PCR at the time of renal biopsy in patients with 30≤eGFR<60 and eGFR≥60 ml/min/1.73m^2^, respectively.

Adjusted for age, gender, MAP, eGFR, BMI and the Oxford Classification MEST- C markers.

**Figure 3:**
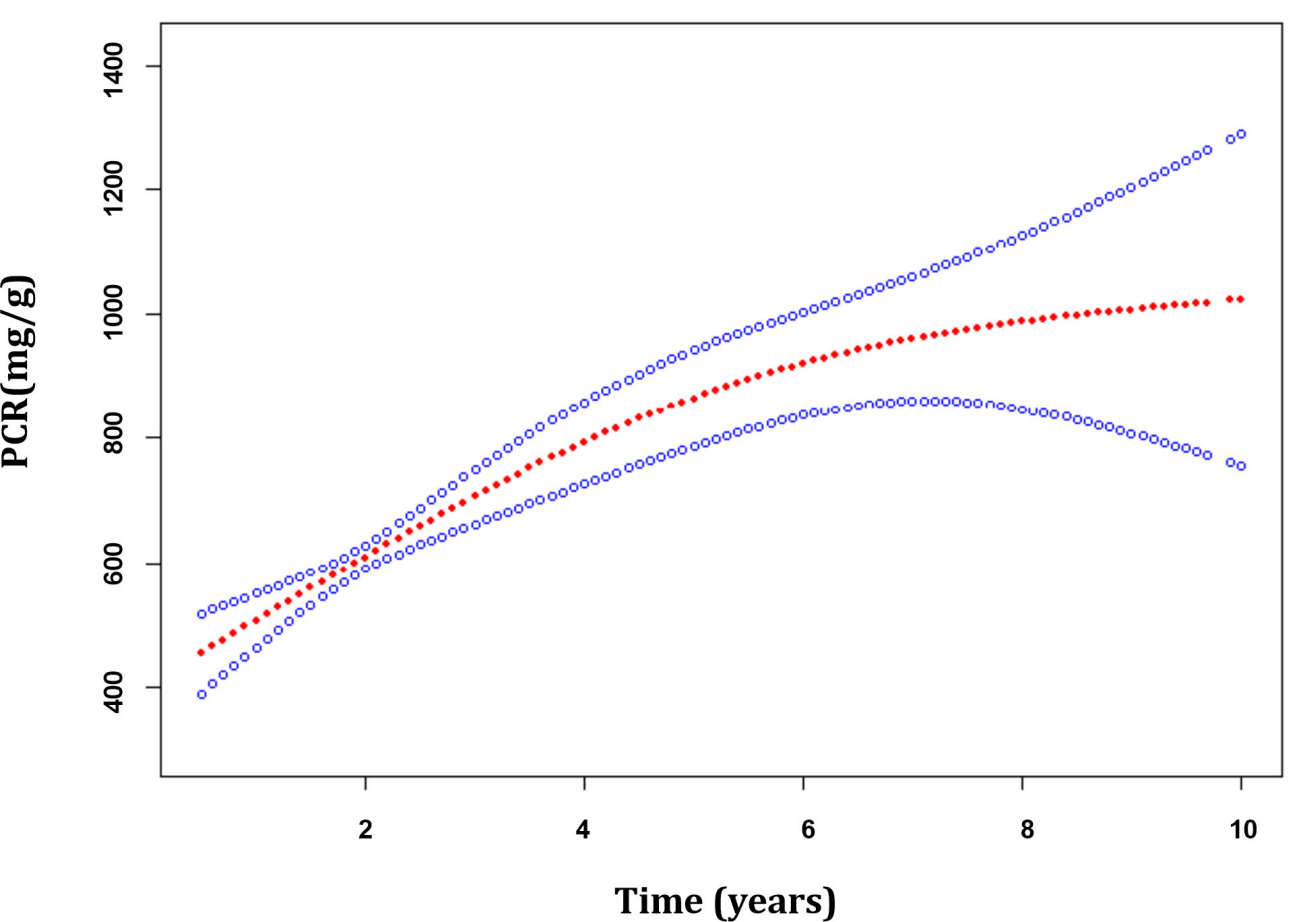
The trajectories of the proteinuria/creatinine ratio in patients with immunoglobulin A nephropathy. Estimates were made from mixed-effect models adjusted for age, gender, MEST-C score, mean arterial pressure, estimated glomerular filtration rate, body mass index and treatments. The red line indicates the estimated value of the proteinuria/creatinine ratio, and the blue line represents the 95% confidence interval for the mean.

**Figure 4:**
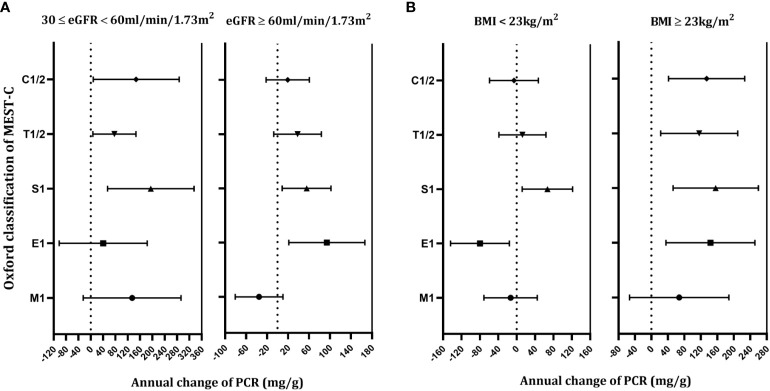
Correlation between the Oxford Classification MEST-C score and the annual changes in proteinuria/creatinine ratio in immunoglobulin A nephropathy patients with estimated glomerular filtration rate ≥30, <60, and ≥60 ml/min/1.73m^2^ **(A)**, body mass index <23 and **≥**23kg/m^2^ **(B)**, respectively. Estimates were made from mixed-effect models adjusted for age, gender, MEST-C score, mean arterial pressure, estimated glomerular filtration rate, body mass index and treatments. Estimates (95% confidence interval) are shown in mg/g per year for each Oxford Classification score.

The median duration of follow-up starting from renal biopsy was 2.60 years (IQR, 1.30–5.05 years). A total of 1,479 PCR measurements, taken from the 358 patients, were available for further analysis. Generalized additive mixed models (GAMMs) were used to evaluate the associations of the MEST-C scores and longitudinal changes in PCR. After adjusting for covariates including age, gender, MAP, eGFR, BMI, MEST-C score, and treatments (RASi alone, RASi in combination with CSs/ISs, CSs/ISs alone, no RASi and CSs/ISs) in the models, it was found that for every additional year, the PCR increased by 68.82 mg/g (95% confidence interval [CI], 44.63–93.02 mg/g; p < 0.001) on average in the entire cohort (**Figure 3**). In patients with renal insufficiency, compared with those without relative lesions, S1 (194.96; 95% CI, 54.50–335.43 mg/g per year; p = 0.007) was associated with the fastest PCR increase. Additionally, C1/2 (147.59; 95% CI, 8.32–286.86 mg/g per year; p = 0.038), and T1/2 (77.04; 95% CI, 7.18–146.89 mg/g per year; p = 0.031) demonstrated a more rapid PCR rise. However, no associations were found between M1, E1 and elevated PCR in the adjusted models (p > 0.05). Among patients with normal kidney function, similar results were found in those with S1 (55.46; 95% CI, 8.93–101.99 mg/g per year; p = 0.020) and E1 (94.02; 95% CI, 21.47–166.58 mg/g per year; p = 0.011) lesions, but not in those with M1, T1/2 and C1/2 lesions (p > 0.05) (**Table 3**; **Figure 4A**).

**Table 3:** Association of the Oxford Classification score MEST-C with the changes in PCR according to 30≤eGFR<60 and eGFR ≥60 ml/min/1.73m^2^.

Oxford Classification	30≤eGFR<60 ml/min/1.73m^2^		eGFR≥60 ml/min/1.73m^2^	
	Beta (95% CI)	*P* value	Beta (95% CI)	*P* value
Time, yr	120.75 (54.05,187.46)	<0.001	44.88 (26.84, 62.91)	<0.001
M1	223.33 (-600.29,1046.94)	0.597	-360.98 (-794.69, 72.73)	0.104
E1	-292.49 (-971.86, 386.87)	0.402	549.08 (44.77, 1053.39)	0.034
S1	-79.74 (-708.13, 548.66)	0.804	150.76 (-295.66, 597.17)	0.509
T1/2	149.59 (-221.72, 520.90)	0.431	236.14 (-446.22, 918.50)	0.498
C1/2	144.87 (-471.51, 761.26)	0.647	41.76 (-370.49, 454.02)	0.843
Time×M1	134.48 (-24.07, 293.04)	0.097	-35.31(-80.84, 10.21)	0.129
Time×E1	40.71(-102.05,183.46)	0.577	94.02 (21.47,166.58)	0.011
Time×S1	194.96 (54.50, 335.43)	0.007	55.46 (8.93, 101.99)	0.020
Time×T1/2	77.04 (7.18,146.89)	0.031	38.17 (-7.27, 83.61)	0.100
Time×C1/2	147.59 (8.32, 286.86)	0.038	19.41(-21.93, 60.75)	0.358

Generalized additive mixed models (GAMMs) were used to investigate the nonlinear fixed effects of M, E, S, T, and C associations with the longitudinal changes in PCR in patients with 30≤eGFR<60 and eGFR≥60 ml/min/1.73m^2^, respectively. Estimates are in mg/g per 1 SD increment in biomarker.

Adjusted for age, gender, MAP, eGFR, BMI, treatments and the Oxford Classification MEST-C markers (GAMM method included the initial PCR as a covariate automatically).

In order to examine the impact of body size on proteinuria, we then classified patients into underweight/normal weight and overweight/obesity groups based on whether their BMI was greater than 23 kg/m^2^. The results indicated that, in patients with underweight/normal weight, those with E1 [-79.67; 95% CI, (-143.68) – (-15.66) mg/g per year; p = 0.015] were associated with a greater PCR declined, while those with S1(67.02; 95% CI, 12.23 – 121.81 mg/g per year; p = 0.017) lesions were correlated with faster PCR increase. However, no correlations between M1, T1/2, C1/2, and PCR change were discovered in the adjusted models (p > 0.05). In patients with overweight/obesity, E1 (143.34; 95% CI, 35.30–251.38 mg/g per year; p = 0.010), S1 (156.09; 95% CI, 52.41–259.77 mg/g per year; p = 0.003), T1/2 (116.04; 95% CI, 22.58–209.51 mg/g per year; p = 0.015), and C1/2 (134.03; 95% CI, 41.73–226.32 mg/g per year; p = 0.005) lesions were associated with noticeably quicker PCR increase, but not in individuals who had M1 lesions. (**Table 4**; **Figure 4B**).

**Table 4:** Association of the Oxford Classification score MEST-C with the changes in PCR according to BMI**<**23kg/m^2^ and BMI≥23kg/m^2^.

Oxford Classification	BMI<23kg/m^2^		BMI≥23kg/m^2^	
	Beta (95% CI)	*P* value	Beta (95% CI)	*P* value
Time, yr	27.93 (2.51, 53.35)	0.121	96.52 (49.67, 143.36)	<0.001
M1	-474.26 (-1070.61, 122.10)	0.121	158.16 (-287.56, 603.88)	0.488
E1	567.91 (-61.20, 1197.02)	0.079	-4.93 (-473.56, 463.69)	0.984
S1	112.65 (-424.80, 650.10)	0.682	115.98 (-305.00, 536.96)	0.590
T1/2	255.15 (-446.38, 956.68)	0.477	518.43 (-3.04, 1039.89)	0.054
C1/2	173.67 (-337.15, 684.50)	0.506	-47.10 (-473.41, 379.21)	0.829
Time×M1	-13.04 (-71.05, 44.96)	0.660	67.33 (-53.29, 187.96)	0.274
Time×E1	-79.67 (-143.68, -15.66)	0.015	143.34 (35.30, 251.38)	0.010
Time×S1	67.02 (12.23, 121.81)	0.017	156.09 (52.41, 259.77)	0.003
Time×T1/2	12.62 (-38.56, 63.80)	0.629	116.04 (22.58, 209.51)	0.015
Time×C1/2	-5.76 (-58.93, 47.41)	0.832	134.03 (41.73, 226.32)	0.005

Generalized additive mixed models (GAMMs) were used to investigate the nonlinear fixed effects of M, E, S, T, and C associations with the longitudinal changes in PCR in patients with BMI**<**23kg/m^2^ and BMI≥23kg/m^2^, respectively. Estimates are in mg/g per 1 SD increment in biomarker.

Adjusted for age, gender, MAP, eGFR, BMI, treatments and the Oxford Classification MEST-C markers (GAMM method included the initial PCR as a covariate automatically).

## Methods

### Study design and patients

This was a single-center retrospective cohort study that included patients with biopsy-proven primary IgAN as recorded in the IgAN Database of Shenzhen Second People’s Hospital between January 1, 2011, and May 31, 2021. Patients with a secondary cause of IgAN, such as Henoch–Schönlein purpura, systemic lupus erythematosus, or chronic liver disease, were excluded, as were those with missing baseline PCR or renal pathology data for the Oxford Classification, a baseline eGFR <30 ml/min/1.73 m^2^, missing follow-up PCR measurements, or a follow-up time <0.5 years (**Figure 1**).

This study was approved by the Medical Ethics Committee of Shenzhen Second People’s Hospital (No. 20211108001-FS01) and conducted ethically in accordance with the World Medical Association Declaration of Helsinki.

### Outcomes

The outcomes consisted of estimated annual changes in PCR after renal biopsy. If patients developed eGFR <15 ml/min/1.73 m^2^, underwent kidney transplantation, hemodialysis, or peritoneal dialysis, or transferred to another center, these were considered to be censored events. The remaining patients were followed up with until December 31, 2021.

### Covariates

The demographic and clinicopathologic data at biopsy were gathered from the hospital’s IgAN database. The baseline covariates analyzed in the multivariate linear regression models included age, gender, MAP (calculated as 1/3 × systolic blood pressure + 2/3 × diastolic blood pressure), BMI, eGFR (the 2009 Chronic Kidney Disease Epidemiology Collaboration creatinine equation (28) was used for calculation), the Oxford Classification MEST-C score. Except for the covariates adjusted in the multivariate linear regression models, the treatments (RASi alone, RASi in combination with CSs/ISs, CSs/ISs alone, no RASi and CSs/ISs) were also included in the multivariable mixed models (the GAMM (29) automatically included the initial PCR as a covariate). As the numbers of patients with C2 (N = 22) and T2 (N = 9) lesions were low, patients with C present ≥1 glomerulus (C1) (N = 170) and C2 lesions were combined into a C1/2 group (N = 192), and those with T 26%–50% (T1) (N = 75) and T2 lesions were also grouped together for analysis as a T1/2 group (N = 84). According to the World Health Organization classification for Asian population, patients were categorized as underweight, normal weight, overweight, and obesity, using the thresholds of <18.5, 18.5–22.9, 23.0–24.9, and ≥25.0 kg/m^2^, respectively. In order to investigate the impact of body size on proteinuria, we categorized patients into underweight/normal weight and overweight/obesity groups based on whether their BMI was greater than 23 kg/m^2^.

Light immunofluorescence and electron microscopy were used for renal biopsy specimen examination, and the histopathology was graded based on the revised Oxford Classification system as follows: M <0.5 (M0) or M1; E absent (E0) or E1; S absent (S0) or S1; T ≤25% (T0), T1, or T2; and C absent (C0), C1, or C2 (2). The renal biopsy results were reviewed independently by two renal pathologists from Guangzhou Kingmed Center for Clinical Laboratory, and if there was a difference of opinion, a final pathological diagnosis was made by a third senior pathologist.

### Statistical analyses

Quantitative variables with normal distribution were expressed as the mean ± standard deviation, and variables with skewed distribution were expressed as the median (quartile), and compared using the t-test or Mann-Whitney test. Categorical variables were expressed as the frequency (percentage) and compared using the chi-squared test or Fisher’s exact test. In addition, a generalized additive model (GAM) and a two-piecewise linear regression model were applied to identify the non-linear relationship and calculate the threshold effect between eGFR and proteinuria.

Multivariate linear regression was applied to identify the associations of the Oxford Classification of MEST-C scores and PCR in patients with 30≤eGFR<60 ml/min/1.73m^2^ and eGFR≥60 ml/min/1.73m^2^ respectively, the independent variables were the Oxford Classification of MEST-C scores, and baseline PCR at the time of renal biopsy was as dependent variable. Age, gender, MAP, BMI, eGFR, the Oxford Classification MEST-C scores were adjusted in the multivariable linear regression models.

Graphical examination of the PCR trajectories showed curvilinear changes in the results. Generalized additive mixed models were used to examine the fixed effects of the Oxford Classification of MEST-C associations with longitudinal changes in PCR (29) in patients with 30≤eGFR<60 ml/min/1.73m^2^ and eGFR≥60 ml/min/1.73m^2^, and in patients with BMI <23kg/m^2^ and BMI**≥**23kg/m^2^, respectively. The dependent variable (PCR) was analyzed at the baseline visit and during all follow-up visits, whereas the independent variables were only evaluated at the baseline visit in these models (Oxford Classification MEST-C score). Age, gender, MAP, BMI, eGFR, the Oxford Classification MEST-C score, and the treatments were adjusted in the multivariable models to determine whether the effects of MEST-C on the changes in PCR were independent. The interaction term between a fixed-effect variable (M, E, S, T, or C) and time in these mixed-effect regression models was used to assess whether the variable was associated with the longitudinal changes in PCR.

All analyses were performed using the statistical software packages R (the R Foundation, Vienna, Austria), EmpowerStats (X&Y Solutions, Inc., Boston, MA, U.S.A.), and GraphPad Prism 8 (GraphPad Software Inc, La Jolla, CA, U.S.A.). A p-value <0.05 was considered statistically significant.

## Discussion

The Oxford Classification system has been well accepted as a means of evaluating the severity of kidney lesions and predicting renal outcomes in patients with IgAN (30). However, the relationships between Oxford Classification scores and clinical remission are still unclear, and this is the first study to comprehensively evaluate the relationship between Oxford Classification MEST-C scores and longitudinal changes in proteinuria. At the time of renal biopsy, it was found that the active lesions including E1 and C1/2 were independently associated with increased proteinuria in IgAN patients. Using repeated PCR measurement data, our results showed that patients with E1, S1, T1/2 and C1/2 lesions demonstrated faster proteinuria increases than patients without such lesions, and those with renal disfunction or overweight/obese patients with relative lesions experienced noticeably quicker PCR raise.

The S lesion has been well recognized for its predictive effect of renal outcomes in patients with IgAN (3, 4, 6–8, 13), and using repeated eGFR measurement data for analysis, a previous study also showed that patients with S lesions display steeper eGFR declines than patients without such lesions (19). In the present study, compared with patients without S lesions, those with S1 lesions were not demonstrated to be associated with increased proteinuria at the time of renal biopsy. However, in patients with renal insufficiency, those with S1 lesions had a notably faster PCR increase (194.96 mg/g per year) during follow-up period. The associations were also demonstrated in patients with normal kidney function although there was a decrease in growth (55.46 mg/g per year). This happened although 79.81% of patients with S1 lesions were treated with RASi, 47.7% of them were treated with CSs/ISs. These results suggested that the effect of segmental glomerular sclerosis on renal damage is persistent but might be partially modifiable by CSs/ISs and/or RASi, especially in patients with normal kidney function. Although it was seen that S lesions are risk factors modifiable by immunosuppressive treatment in the patients, there were differences in the eGFR between the treated and untreated patients (31, 32). A meta-analysis of five studies with 637 patients showed that S lesions significantly predict steroid resistance (33), and moreover, a re-evaluation of Oxford Classification scores on repeated biopsies showed that S lesions do not improve after immunosuppressive treatments (17, 34, 35). Coppo et al. (36) used the original cohort from the Validation Study of the Oxford Classification for IgAN (VALIGA) with an extended follow-up period of 35 years for analysis, and they found that S1 lesions are still independently correlated with renal outcomes. Thus, the results of this study as well as previous studies suggest that S1 lesions might be responding poorly to currently available treatments.

It has been shown that one of the strongest predictive factors for renal outcomes in patients with IgAN is T lesions (2), and it has been shown that compared with patients without such lesions, patients with T2 lesions experience a substantially faster eGFR decline per year (19). Similar to S lesions, the present study did not find significant association between T lesions and raised proteinuria levels at the time of renal biopsy. But we found that T lesions were moderately associated with longitudinal proteinuria increases in IgAN patients with renal disfunction. However, this relationship could not be verified in patients with normal kidney function. Although the correlations between tubulointerstitial lesions and proteinuria were not so strong as the correlations between glomerular lesions and proteinuria, one study noted that proteinuria in IgAN might not only result from active lesions but also from sclerotic glomerular lesions with hyperfiltration and tubular damage (37). Bellur et al. (38) used the cohort from VALIGA to investigate the use of the Oxford Classification of IgAN in 1,147 patients from 13 European countries. They found that all glomerular lesions (M, E, C, and S) were independently associated with the decision to administer CS/IS therapy, except tubulointerstitial lesions. However, in the VALIGA extended follow-up study (36), T1/2 lesions were found to have a predictive value decades after renal biopsy. It was also found that T lesions in association with proteinuria are valuable prognostic markers for the progression of IgAN (39, 40). From the analysis of the VALIGA propensity score-matched cohort (31), the percentage of patients with T1 lesions who ended the follow-up with proteinuria <1 g/day was significantly higher in the group treated with CSs/ISs than in that receiving ACEIs/ARBs alone. Although studies have shown that T1/2 lesions seem to persist and even deteriorate, some T1 lesions improve and change to T0 after treatments (34, 35). Moreover, T1/2 lesions involving >25% of the renal biopsy area represent medium to severe damage, and they are more common in patients with a marked decline in renal function. In this study, T1/2 lesions accounted for 24.3% and 6.7% of patients with eGFR <60 ml/min/1.73m^2^ and eGFR ≥60 ml/min/1.73m^2^, respectively. And 77.38% of patients with T1/2 lesions took RASi and 59.53% of them took CSs/ISs after renal biopsy. The insignificant results in patients with normal kidney function may largely have been due to the small number of patients with T1/2 lesions in this sample, and further studies are still needed to confirm this hypothesis. For patients with eGFR ≥30 ml/min/1.73 m^2^ who have T lesions, especially T1 lesions in the early stages, the disease might be partially reversible, and more effective therapies targeted toward these lesions might help improve the renal prognosis.

Unlike S and T lesions, E lesions are known to be active, and inconsistent results for their predictive role in renal outcomes have been reported (3, 5–18, 41, 42). The differences are regarded to be confounded by an immunosuppressant bias (17, 31, 33, 35, 43). The present study showed that patients with E1 lesions were associated with increased proteinuria at the time of renal biopsy. Moreover, the patients with E1 lesions exhibited a faster proteinuria rise (94.02 mg/g per year) than those without such lesions in patients with normal kidney function, but not in those with kidney function impairment. Among the study patients, 74.02% of those with E1 lesions took RASi, and 64.93% of them took CSs/ISs, and a lower proportion of patients without E receiving CSs/ISs (41.61%). It’s probable that the results were viewed as negligible in part due to the small number of individuals with renal insufficiency and E1 lesions in our sample, and this idea still needs to be tested in more studies. A study conducted by Coppo et al., in which the original VALIGA cohort was used for analysis, with a median follow-up of 4.7 years. It was concluded that an E lesion is a risk factor for developing higher proteinuria levels (4). In a single-center European study investigating patients with IgAN who did not undergo CS/IS treatment, irrespective of the clinical features at renal biopsy, E1 was the best predictor of poor renal survival at the six-year follow-up (44). Jullien et al. examined repeated kidney biopsies and found that E1 lesions discovered in the first renal biopsy were persistent in 62% of patients in the second renal biopsy after a median time of 5.4 years and that the persistent E1 lesions were associated with poor renal survival (35). Thus, further studies are still needed to confirm whether E1 lesions are satisfactorily treatable with current therapies, especially immunosuppressants.

The prognostic role of C lesions in renal decline progression was less concordant and reproducible and were not included in the original Oxford Classification score system. A working subgroup of the IgAN Classification Working Group addressed crescents as a potential predictor for renal outcomes in 3,096 patients with IgAN, who were assembled from four retrospective studies (Haas et al. (18), Oxford (3, 45), and VALIGA (4)) and two large Asian databases (5, 11). They discovered that C1 lesions only predicted renal outcomes in individuals not receiving immunosuppressive therapy, whereas C2 lesions indicated poor renal outcomes in both immunosuppressed patients and those who were not. In this study, we discovered that patients with C1/2 lesions had higher proteinuria at the time of renal biopsy and that they also experienced a faster rise in proteinuria (147.59 mg/g per year) than patients without such lesions. These correlations were not observed in patients with normal kidney function. In the current study, 58.6% of patients with C1/2 and 32.03% of patients without related lesions took CSs/ISs, respectively. In our clinical practice, C lesions have been acknowledged as active injuries and are more likely to be treated with immunosuppressive medications. These findings would suggest that current therapies, particularly immunosuppressants, may work better on patients with normal renal function than on those with impaired renal function for treating C lesions. Two studies of repeated biopsies showed that patients with cellular/fibrocellular crescents displayed significant improvement after immunosuppressant use (17, 43). Of these two studies, Shen et al. found that after immunosuppressive therapy, 10% of patients with C lesions on the first biopsy had vanished, 5% had gotten worse, 65% had reversed, and 20% had remained in the second biopsy (17). Another study found no statistically significant changes between the first and second biopsy with respect to C lesions (16% vs. 11%) (35). However, neither the difference in renal function nor the severity of the crescents (i.e., C1 or C2), which might influence the treatment response in IgAN patients, were examined in these trials. Therefore, further research is still needed to confirm the effectiveness of the present medicines for treating C lesions.

Overweight/obese is considered as a strong predictor of new onset of chronic kidney disease and progression of ESRD (46). Although the exact mechanism is still unclear, overweight/obese have been linked to the induction of proteinuria, which might be through physical compression of the kidney or alteration of glomerular hyperfiltration, along with inflammation, oxidative stress, lipotoxicity, as well as activation of the renin-angiotensin-aldosterone system (RAAS) and mineralocorticoid receptor (47–49). Similarly, the current study demonstrated a correlation between overweight/obese patients with lesions on the E1 (143.34 mg/g per year), S1 (156.09 mg/g per year), T1/2 (116.04 mg/g per year), and C1/2 (134.03 mg/g per year) and considerably quicker PCR rise. Interestingly, for patients who were underweight/normal weight, E1 (-79.67 mg/g per year) lesions were associated with faster proteinuria decline during the follow-up period, indicating a better treatment response for these patients. A RCT recruiting of patients with nephropathy of diabetic or nondiabetic cause showed that weight loss of 4%, there was a 30% decrease in proteinuria, and with further 6–10% reduction in weight the proteinuria decreased by > 70% (50, 51). These findings imply that weight loss may improve renal prognosis in overweight/obese patients by lowering urine protein and/or by other mechanisms. However, as IgAN patients were not included in the previous study, the effects of weight loss on long-term urine protein levels and renal function in IgAN patients who are overweight/obese, is still need to be further investigated.

The greatest strength of the current study was the use of repeated PCR measurements, which made it possible to track longitudinal changes in proteinuria according to the Oxford Classification score. However, this study also had some limitations. As a retrospective study, it was only possible to obtain the treated and untreated data from the electronic medical records, and it was not possible to preclude patients with poor medicine compliance who claimed to comply with the medical advice. In addition, this study only separately analyzed patients with one of the MEST-C lesions, but patients often have more than two pathological changes. Further studies with a larger sample and subgroup analysis with different combinations of Oxford Classification scores are needed. Moreover, research on microangiopathic lesions, which have been proven to predict renal failure progression, needs to be included (52). Finally, the follow-up time of this study was relatively short, and only data from one center were analyzed. Thus, these results must be verified in other centers and among other ethnic groups and with longer follow-up times.

## Conclusion

In summary, Oxford Classification scores E1 and C1/2 were associated with increased proteinuria at the time of renal biopsy, using repeated PCR measurement data during follow-up visits for analysis, it was found that S1, E1, T1/2, and C1/2 were associated with faster proteinuria increases in patients with IgAN, especially in those with renal insufficiency or overweight/obesity. Thus, the Oxford Classification score might be valuable for developing individual treatment therapies since the findings suggest that currently available treatments are unsatisfactory for treating these lesions successfully, and weight loss may be helpful for lowering urine protein in overweight/obese patients.

## Data availability statement

The original contributions presented in the study are included in the article/**Supplementary Material**. Further inquiries can be directed to the corresponding author.

## Ethics statement

The studies involving human participants were reviewed and approved by The Second People’s Hospital of Shenzhen. The patients/participants provided their written informed consent to participate in this study.

## Author contributions

Conception and design of the research: R-CX and Q-JW. Acquisition of data: YL, Y-NC, FT and QX. Analysis and interpretation of the data: J-YG, TC and YX. Statistical analysis: H-YS, X-JC and M-JG. Obtaining financing: R-CX, Q-JW and YX. Writing of the manuscript: R-CX and J-YG. Critical revision of the manuscript for intellectual content: X-LC and Q-JW. All authors contributed to the article and approved the submitted version.

## Supplementary material

Supplementary Figure 1 The relationship between estimated glomerular filtration rate and proteinuria/creatinine ratio in patients with immunoglobulin A nephropathy. A non-linear relationship between estimated glomerular filtration rate and proteinuria/creatinine ratio was detected after adjusting for age, gender, mean arterial pressure, body mass index and the Oxford Classification MEST-C score.
