# Supplementary figures and images for: A mixed-method evaluation of the relationship between Oxford classification scores and longitudinal changes in proteinuria in patients with immunoglobulin A nephropathy
Source: Front Endocrinol (Lausanne). 2023 Jan 10;13:890900. doi: 10.3389/fendo.2022.890900 (PMC9871483; doi:10.3389/fendo.2022.890900)

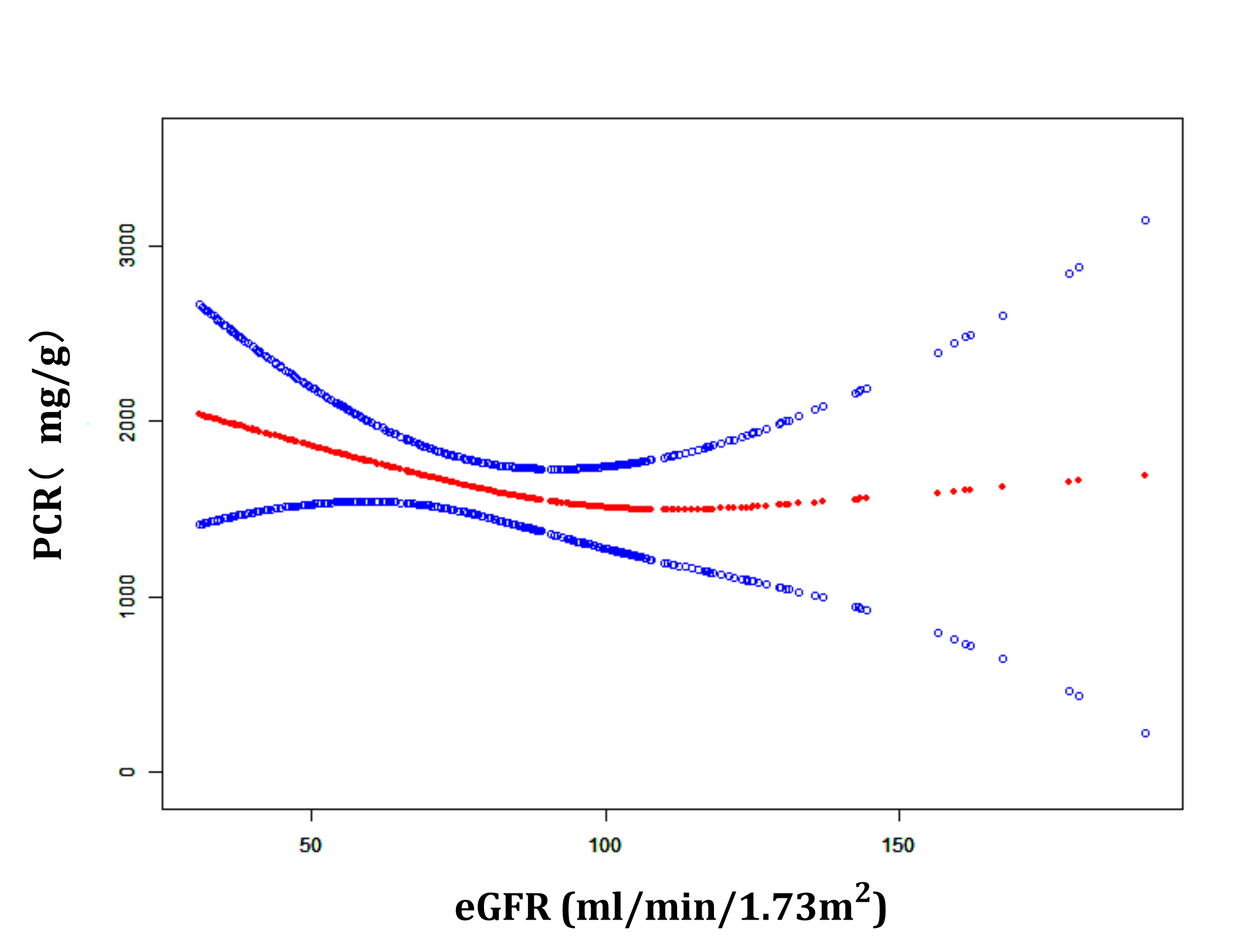

Supplement: Supplementary Figure 1 — The relationship between estimated glomerular filtration rate and proteinuria/creatinine ratio in patients with immunoglobulin A nephropathy. A non-linear relationship between estimated glomerular filtration rate and proteinuria/creatinine ratio was detected after adjusting for age, gender, mean arterial pressure, body mass index and the Oxford Classification MEST-C score. [file Image_1.tif]
