# Supplementary material for: A mixed-method evaluation of the relationship between Oxford classification scores and longitudinal changes in proteinuria in patients with immunoglobulin A nephropathy
Source: Front Endocrinol (Lausanne). 2023 Jan 10;13:890900. doi: 10.3389/fendo.2022.890900 (PMC9871483; doi:10.3389/fendo.2022.890900)
Supplement: Supplementary file 2 [file Table_1.docx]

**Supplementary Table 1**. **Baseline characteristics and pathological features of IgAN patients accroding to M lesions.**

| **Variables** | **M0**  **(n=82)** | **M1**  **(n=272)** | ***P* value** |
| --- | --- | --- | --- |
| Age, yr | 36.91 ± 9.54 | 34.22 ± 9.29 | 0.009 |
| Male, n (%) | 38 (46.34%) | 138 (50.55%) | 0.504 |
| BMI, kg/m^2^ | 23 ± 3.44 | 22.61 ± 3.38 | 0.307 |
| BMI group, n (%) |  |  |  |
| Underweight/normal weight | 41 (51.90%) | 149 (60.32%) | 0.186 |
| Overweight/obesity | 38 (48.10%) | 98 (39.68%) |  |
| SBP, mmHg | 128.30 ± 19.23 | 127.42 ± 18.70 | 0.815 |
| DBP, mmHg | 82.73 ± 10.82 | 82.25 ±13.23 | 0.471 |
| MAP, mmHg | 96.94 ± 12.61 | 96.33 ±14.07 | 0.604 |
| Hemoglobin, g/L | 130.26 ± 18.63 | 128.39 ± 20.14 | 0.602 |
| Albumin, g/L | 39.60 ± 5.30 | 39.08 ± 5.48 | 0.648 |
| Total cholesterol, mmol/L | 4.79 ±1.38 | 4.82 ± 1.32 | 0.787 |
| Triglyceride, mmol/L | 1.59 ± 1.13 | 1.59 ± 1.30 | 0.425 |
| Uric acid, umol/L | 396.44 ± 103.50 | 406.06 ± 113.51 | 0.691 |
| eGFR, ml/min/1.73m^2^ | 85.19  (65.19, 100.99) | 80.08  (55.92, 101.81) | 0.410 |
| Proteinuria/creatinine ratio, mg/g | 842.50  (569.00, 1512.50) | 1118.50  (645.75, 1812.00) | 0.170 |
| Hematuria, n (% ) | 54(65.85%) | 198(72.79%) | 0.243 |
| Follow up time, yr | 3.10 (2.30, 5.10) | 2.20 (1.20, 5.00) | 0.052 |
| Treatments, n (%) |  |  | 0.170 |
| RASi alone, n (%) | 32 (42.11%) | 113 (42.97%) |  |
| RASi + CSs/ISs, n (%) | 19 (25.00%) | 93 (35.36%) |  |
| CSs/ISs alone, n (%) | 14 (18.42%) | 33 (12.55%) |  |
| No RASi and CSs/ISs, n (%) | 11 (14.47%) | 24 (9.13%) |  |
| Oxford Classification, n (%) |  |  |  |
| Mesangial hypercellularity (M1) | 0 | 272 (100.00%) | - |
| Endocapillary hypercellularity (E1) | 14 (17.28%) | 66 (24.18%) | 0.193 |
| Segmental glomerulosclerosis (S1) | 21 (25.61%) | 96 (35.16%) | 0.106 |
| Tubular atrophy/interstitial fibrosis (T1/2) | 13 (15.85%) | 72 (26.37%) | 0.050 |
| Cresent (C1/2) | 36 (45.00%) | 156 (57.78%) | 0.044 |

Data presented as mean ± SD, median (25th, 75th) or number (percent).

Abbreviations: SBP, systolic blood pressure; DBP, diastolic blood pressure; MAP, mean artery pressure; eGFR, estimated glomerular filtration rate; BMI, body mass index; RASi, renin-angiotensin system inhibitors; CSs/ISs, corticosteroids and (or) immunosuppressants.

**Supplementary Table 2**. **Baseline characteristics and pathological features of IgAN patients accroding to E lesions.**

| **Variables** | **E0**  **(n=274)** | **E1**  **(n=80)** | ***P* value** |
| --- | --- | --- | --- |
| Age, yr | 34.25 ± 8.74 | 36.83 ± 11.21 | 0.073 |
| Male, n (%) | 137 ( 49.82%) | 38 (47.50%) | 0.715 |
| BMI, kg/m^2^ | 22.59 ± 3.32 | 23.11 ± 3.64 | 0.259 |
| BMI group, n (%) |  |  | 0.467 |
| Underweight/normal weight | 153 (59.30%) | 37 (54.41%) |  |
| Overweight/obesity | 105 (40.70%) | 31 (45.59%) |  |
| SBP, mmHg | 126.28 ± 17.26 | 132.29 ± 22.84 | 0.123 |
| DBP, mmHg | 81.82 ± 11.61 | 84.46 ± 15.80 | 0.352 |
| MAP, mmHg | 95.67 ± 12.43 | 99.40 ± 17.27 | 0.258 |
| Hemoglobin, g/L | 128.75 ± 20.41 | 128.85 ± 17.38 | 0.738 |
| Albumin, g/L | 39.52 ± 5.41 | 38.03 ± 5.41 | 0.035 |
| Total cholesterol, mmol/L | 4.72 ± 1.30 | 5.13 ± 1.40 | 0.025 |
| Triglyceride, mmol/L | 1.52 ± 1.15 | 1.82 ± 1.57 | 0.106 |
| Uric acid, umol/L | 405.22 ± 110.74 | 399.32 ± 113.36 | 0.620 |
| eGFR, ml/min/1.73m^2^ | 82.07  (59.52, 101.66) | 76.15  (54.64, 100.36) | 0.326 |
| Proteinuria/creatinine ratio, mg/g | 916.50  (545.50, 1617.50) | 1426.50  (864.50, 2311.75) | <0.001 |
| Hematuria, n (% ) | 191 (69.45%) | 61 (76.25%) | 0.238 |
| Follow up time, yr | 2.70 (1.40, 4.90) | 2.30 (1.20, 5.78) | 0.794 |
| Treatments, n (%) |  |  | 0.003 |
| RASi alone, n (%) | 125 (47.71%) | 20 (25.97%) |  |
| RASi + CSs/ISs, n (%) | 75 (28.63%) | 37 (48.05%) |  |
| CSs/ISs alone, n (%) | 34 (12.98%) | 13 (16.88%) |  |
| No RASi and CSs/ISs, n (%) | 28 (10.69%) | 7 (9.09%) |  |
| Oxford Classification, n (%) |  |  |  |
| Mesangial hypercellularity (M1) | 207 (75.55%) | 66 (82.50%) | 0.193 |
| Endocapillary hypercellularity (E1) | 0 | 80 (100.00%) | - |
| Segmental glomerulosclerosis (S1) | 88 (32.00%) | 30 (37.50%) | 0.358 |
| Tubular atrophy/interstitial fibrosis (T1/2) | 60 (21.82%) | 24 (30.00%) | 0.130 |
| Cresent (C1/2) | 142 (52.59%) | 51 (63.75%) | 0.078 |

Data presented as mean ± SD, median (25th, 75th) or number (percent).

Abbreviations: SBP, systolic blood pressure; DBP, diastolic blood pressure; MAP, mean artery pressure; eGFR, estimated glomerular filtration rate; BMI, body mass index; RASi, renin-angiotensin system inhibitors; CSs/ISs, corticosteroids and (or) immunosuppressants.

**Supplementary Table 3**. **Baseline characteristics and pathological features of IgAN patients accroding to S lesions.**

| **Variables** | **S0**  **(n=238)** | **S1**  **(n=118)** | ***P* value** |
| --- | --- | --- | --- |
| Age, yr | 34.71 ± 9.48 | 35.26 ± 9.29 | 0.640 |
| Male, n (%) | 125 (52.52%) | 51 (42.86%) | 0.085 |
| BMI, kg/m^2^ | 22.65 ± 3.26 | 22.82 ± 3.64 | 0.863 |
| BMI group, n (%) |  |  | 0.827 |
| Underweight/normal weight | 124 (57.67%) | 66 (58.93%) |  |
| Overweight/obesity | 91 (42.33%) | 46 (41.07%) |  |
| SBP, mmHg | 126.89 ± 18.89 | 128.97 ± 18.51 | 0.346 |
| DBP, mmHg | 81.77 ± 12.77 | 83.60 ± 12.44 | 0.109 |
| MAP, mmHg | 95.84 ± 13.79 | 97.73 ± 13.49 | 0.135 |
| Hemoglobin, g/L | 129.79 ± 19.66 | 126.77 ± 19.87 | 0.302 |
| Albumin, g/L | 39.32 ± 5.72 | 38.85 ± 4.85 | 0.140 |
| Total cholesterol, mmol/L | 4.67 ± 1.16 | 5.11 ± 1.58 | 0.009 |
| Triglyceride, mmol/L | 1.48 ± 1.22 | 1.82 ± 1.31 | <0.001 |
| Uric acid, umol/L | 391.06 ± 104.10 | 428.59 ± 120.14 | 0.009 |
| eGFR, ml/min/1.73m^2^ | 87.53  (63.71, 103.83) | 70.93  (53.20, 90.86) | <0.001 |
| Proteinuria/creatinine ratio, mg/g | 879.00  (505.00, 1585.00) | 1395.50  (787.75, 2230.25) | <0.001 |
| Hematuria, n (% ) | 175 (73.53%) | 79 (66.39%) | 0.16 |
| Follow up time, yr | 2.70 (1.30, 5.18) | 2.60 (1.50, 4.90) | 0.954 |
| Treatments, n (%) |  |  | 0.439 |
| RASi alone, n (%) | 96 (41.56%) | 50 (45.87%) |  |
| RASi + CSs/ISs, n (%) | 75 (32.47%) | 37 (33.94%) |  |
| CSs/ISs alone, n (%) | 32 (13.85%) | 15 (13.76%) |  |
| No RASi and CSs/ISs, n (%) | 28 (12.12%) | 7 (6.42%) |  |
| Oxford Classification, n (%) |  |  |  |
| Mesangial hypercellularity (M1) | 177 (74.37%) | 96 (82.05%) | 0.106 |
| Endocapillary hypercellularity (E1) | 50 (21.10%) | 30 (25.42%) | 0.358 |
| Segmental glomerulosclerosis (S1) | 0 | 118 (100.00%) | - |
| Tubular atrophy/interstitial fibrosis (T1/2) | 40 (16.81%) | 45 (38.14%) | <0.001 |
| Cresent (C1/2) | 126 (53.16%) | 67 (58.77%) | 0.323 |

Data presented as mean ± SD, median (25th, 75th) or number (percent).

Abbreviations: SBP, systolic blood pressure; DBP, diastolic blood pressure; MAP, mean artery pressure; eGFR, estimated glomerular filtration rate; BMI, body mass index; RASi, renin-angiotensin system inhibitors; CSs/ISs, corticosteroids and (or) immunosuppressants.

**Supplementary Table 4**. **Baseline characteristics and pathological features of IgAN patients accroding to T lesions.**

| **Variables** | **T0**  **(n=271)** | **T1/2**  **(n=84)** | ***P* value** |
| --- | --- | --- | --- |
| Age, yr | 34.65 ± 9.54 | 35.48 ± 8.91 | 0.514 |
| Male, n (%) | 133 (49.08%) | 43 (50.59%) | 0.808 |
| BMI, kg/m^2^ | 22.58 ± 3.27 | 23.10 ± 3.73 | 0.339 |
| BMI group, n (%) |  |  | 0.782 |
| Underweight/normal weight | 144 (58.54%) | 46 (56.79%) |  |
| Overweight/obesity | 102 (41.46%) | 35 (43.21%) |  |
| SBP, mmHg | 125.34 ± 17.66 | 134.92 ± 20.39 | <0.001 |
| DBP, mmHg | 80.80 ± 12.12 | 87.47 ± 13.20 | <0.001 |
| MAP, mmHg | 94.69 ± 12.91 | 102.25 ± 14.66 | <0.001 |
| Hemoglobin, g/L | 129.44 ± 20.01 | 126.90 ± 18.90 | 0.279 |
| Albumin, g/L | 39.62 ± 5.62 | 37.82 ± 4.57 | <0.001 |
| Total cholesterol, mmol/L | 4.71 ± 1.17 | 5.14 ± 1.69 | 0.024 |
| Triglyceride, mmol/L | 1.50 ± 1.24 | 1.89 ± 1.29 | <0.001 |
| Uric acid, umol/L | 391.18 ± 110.28 | 444.07 ± 104.24 | <0.001 |
| eGFR, ml/min/1.73m^2^ | 90.74  (70.95, 104.28) | 52.18  (39.09, 63.40) | <0.001 |
| Proteinuria/creatinine ratio, mg/g | 869.00  (517.00, 1544.50) | 1682.00  (1054.00, 2700.00) | <0.001 |
| Hematuria, n (% ) | 187 (69.00%) | 66 (77.65%) | 0.125 |
| Follow up time, yr | 2.50 (1.30, 4.70) | 3.10 (1.50, 5.80) | 0.062 |
| Treatments, n (%) |  |  | 0.019 |
| RASi alone, n (%) | 120 (46.88%) | 26 (30.95%) |  |
| RASi + CSs/ISs, n (%) | 73 (28.52%) | 39 (46.43%) |  |
| CSs/ISs alone, n (%) | 36 (14.06%) | 11 (13.10%) |  |
| No RASi and CSs/ISs, n (%) | 27 (10.55%) | 8 (9.52%) |  |
| Oxford Classification, n (%) |  |  |  |
| Mesangial hypercellularity (M1) | 201 (74.44%) | 72 (84.71%) | 0.050 |
| Endocapillary hypercellularity (E1) | 56 (20.66%) | 24 (28.57%) | 0.130 |
| Segmental glomerulosclerosis (S1) | 73 (26.94%) | 45 (52.94%) | <0.001 |
| Tubular atrophy/interstitial fibrosis (T1/2) | 0 | 84 (100.00%) | - |
| Cresent (C1/2) | 142 (53.18%) | 51 (60.71%) | 0.226 |

Data presented as mean ± SD, median (25th, 75th) or number (percent).

Abbreviations: SBP, systolic blood pressure; DBP, diastolic blood pressure; MAP, mean artery pressure; eGFR, estimated glomerular filtration rate; BMI, body mass index; RASi, renin-angiotensin system inhibitors; CSs/ISs, corticosteroids and (or) immunosuppressants.

**Supplementary Table 5**. **Baseline characteristics and pathological features of IgAN patients accroding to C lesions.**

| **Variables** | **C0**  **(n=158)** | **C1/2**  **(n=192)** | ***P* value** |
| --- | --- | --- | --- |
| Age, yr | 36.34 ± 9.74 | 33.56 ± 8.91 | 0.006 |
| Male, n (%) | 83 (52.53%) | 89 (46.13%) | 0.231 |
| BMI, kg/m^2^ | 22.97 ± 3.42 | 22.50 ± 3.37 | 0.199 |
| BMI group, n (%) |  |  | 0.708 |
| Underweight/normal weight | 81 (57.04%) | 107 (59.12%) |  |
| Overweight/obesity | 61 (42.96%) | 74 (40.88%) |  |
| SBP, mmHg | 127.95 ± 17.50 | 127.13 ± 19.76 | 0.333 |
| DBP, mmHg | 82.83 ± 11.94 | 81.99 ± 13.24 | 0.217 |
| MAP, mmHg | 96.89 ± 12.73 | 96.07 ± 14.44 | 0.213 |
| Hemoglobin, g/L | 131.89 ± 20.04 | 126.30 ± 18.94 | 0.002 |
| Albumin, g/L | 40.05 ± 5.56 | 38.50 ± 5.22 | 0.001 |
| Total cholesterol, mmol/L | 4.78 ± 1.46 | 4.86 ± 1.22 | 0.273 |
| Triglyceride, mmol/L | 1.50 ± 1.09 | 1.67 ± 1.39 | 0.719 |
| Uric acid, umol/L | 409.84 ± 108.09 | 399.56 ± 114.59 | 0.301 |
| eGFR, ml/min/1.73m^2^ | 79.34  (61.58, 100.80) | 82.43  (57.25, 103.00) | 0.656 |
| Proteinuria/creatinine ratio, mg/g | 803.50  (500.00, 1479.50) | 1297.50  (711.25, 2195.00) | <0.001 |
| Hematuria, n (% ) | 86 (54.43%) | 165 (85.49%) | <0.001 |
| Follow up time, yr | 2.75 (1.30, 4.95) | 2.40 (1.30, 5.40) | 0.988 |
| Treatments, n (%) |  |  | <0.001 |
| RASi alone, n (%) | 91 (59.48%) | 55 (29.57%) |  |
| RASi + CSs/ISs, n (%) | 36 (23.53%) | 75 (40.32%) |  |
| CSs/ISs alone, n (%) | 13 (8.50%) | 34 (18.28%) |  |
| No RASi and CSs/ISs, n (%) | 13 (8.50%) | 22 (11.83%) |  |
| Oxford Classification, n (%) |  |  |  |
| Mesangial hypercellularity (M1) | 114 (72.15%) | 156 (81.25%) | 0.044 |
| Endocapillary hypercellularity (E1) | 29 (18.47%) | 51 (26.42%) | 0.078 |
| Segmental glomerulosclerosis (S1) | 47 (29.75%) | 67 (34.72%) | 0.323 |
| Tubular atrophy/interstitial fibrosis (T1/2) | 33 (20.89%) | 51 (26.42%) | 0.226 |
| Cresent (C1/2) | 0 | 192 (100.00%) | - |

Data presented as mean ± SD, median (25th, 75th) or number (percent).

Abbreviations: SBP, systolic blood pressure; DBP, diastolic blood pressure; MAP, mean artery pressure; eGFR, estimated glomerular filtration rate; BMI, body mass index; RASi, renin-angiotensin system inhibitors; CSs/ISs, corticosteroids and (or) immunosuppressants.

**Supplementary Table 6. The results of two-piecewise linear regression model.**

| **Inflection point of eGFR ml/min/1.73m^2^)** | **Βeta (95% CI)** | ***P* value** |
| --- | --- | --- |
| ＜94.3 | -16.18 (-32.22, -0.14) | 0.048 |
| ≥94.3 | 10.02 (-7.23, 27.27) | 0.255 |

^a^Adjusted for age, gender, MAP, BMI and the Oxford Classification MEST-C markers
